# Same patient, same total knee arthroplasty, different outcomes? A pilot study comparing conventional vs. OMNIBotics Robotic-Assisted HLS Kneetec^TM^ (Corin^®^)

**DOI:** 10.1051/sicotj/2026057

**Published:** 2026-09-01

**Authors:** Galdéric Roblot, Marie Moret, Quentin Seguret, Sofiane Taouli, Bassel Al Hassoun, Olivier Roux, Patrice Mertl, Cecile Laterza, Mike Raimi

**Affiliations:** 1 Service de Chirurgie Orthopédique et Traumatologie Compiegne-Noyon Hospital 8 avenue Henri Adnot Compiegne 60200 France; 2 Service de Chirurgie Orthopédique et Traumatologie CHU Amiens 1 rond-point du Pr Cabrol Picardie 80054 Amiens Cedex France

**Keywords:** Total knee arthroplasty, Adjusted mechanical alignment, Conventional Total Knee Arthroplasty, Robot-Assisted Total Knee Arthroplasty, OMNIBotics

## Abstract

*Introduction*: Total knee arthroplasty (TKA) is a well-established treatment for end-stage knee osteoarthritis. Robotic-assisted TKA (RA-TKA) has emerged over the past decade, enabling patient-specific implantation, precise bone cuts, and intraoperative control of gap balance and alignment. The HLS Kneetec^TM^ is a third condyle posterior stabilized TKA, initially designed for implantation using conventional manual instrumentation (C-TKA), and intended for use with an adjusted mechanical alignment. It can now also be implanted with robotic assistance. This pilot study aimed to evaluate the association between RA-TKA versus C-TKA and clinical outcomes in patients with bilateral knee osteoarthritis. *Materials and methods:* This was a retrospective cohort of patients operated on by primary RA-TKA in a second osteoarthritic knee after contralateral C-TKA. Twenty-two patients constituted their own control group (44 knees). Mean age was 71.9 ± 7.4 years. The mean time between the two procedures was 22 ± 19 months. Primary outcomes measured were limb alignment (hip–knee–ankle (HKA)), range of motion (ROM), and Forgotten Joint Score (FJS) at one year postoperatively. Secondary outcomes included operating time, hospital stay, incision length, blood loss, and radiographic evaluation (femoral mechanical angle (FMA), tibial mechanical angle (TMA), tibial component slope angle (TSA), patellar tilt angle (PTA), and femoral axial rotation angle (FARA)). *Results*: RA-TKA was associated with an HKA angle significantly closer to the target (177.9° vs. 175.3°, *p* = 0.012), with higher flexion (+7°, *p* = 0.032) and a higher FJS (+13.9 points, *p* < 0.001). Operating time was longer in the RA-TKA group (+19.5 min, *p* = 0.004), while hospital stay, blood loss, and incision length did not differ significantly between groups. Radiographically, RA-TKA was associated with reduced femoral valgus (FMA −1.3°, *p* = 0.001), with no significant differences in TMA or TSA. Patellar tilt was similar, whereas femoral external rotation was increased with RA-TKA (+1.1°, *p* = 0.0006). *Conclusion*: In this pilot study, RA-TKA was associated with superior frontal alignment and better patient-reported functional outcomes compared to C-TKA. However, given the inherent temporal confounding of the bilateral sequential design and the small sample size, these findings should be interpreted with caution. Further prospective, adequately powered studies are warranted to confirm these results.

## Introduction

Total knee arthroplasty (TKA) is a well-established treatment for end-stage knee osteoarthritis, with an incidence that continues to rise and is expected to surpass that of total hip arthroplasty by 2030 [1]. Since the 2010s, robotic-assisted TKA (RA-TKA) has become widespread, enabling patient-specific implantation, precise bone resections, intraoperative control of gap balance, and alignment [2]. In our experience, we have used a tri-condylar posterior-stabilized TKA, the HLS Kneetec^TM^ (Corin^®^), for the past decade. This implant has already demonstrated long-term survivorship and good clinical outcomes [3]. We follow an adjusted mechanical alignment technique [4]. Initially, we were using conventional manual instrumentation (C-TKA). But since February 2024, we have adopted the OMNIBotics RA-TKA system to implant the same prosthesis, combining image-free navigation with a robotic ligament tensioner and a robotic cutting guide [5].

Many studies have highlighted the benefits of RA-TKA. Most of this literature focuses on so-called patient-specific alignment strategies such as anatomical, kinematic, inverse kinematic, restricted kinematic, modified kinematic, and functional alignment. By contrast, adjusted mechanical alignment (AMA) is a hybrid technique with previously reported good clinical outcomes [6, 7] has been little studied in the context of RA-TKA. Moreover, most of the existing studies evaluate robotic systems in which ligament balancing is performed through soft-tissue tensioning tests, with the applied force being manually generated by the surgeon. In contrast, the OMNIBotic platform replaces surgeon-dependent laxity assessments with the BalanceBot, a dynamic robotic ligament tensioner that standardizes applied forces.

The purpose was to compare outcomes between knees in patients who underwent bilateral TKA, with one knee implanted using traditional C-TKA and the other using RA-TKA while using the same implant and surgical technique for both knees. We hypothesized that RA-TKA would lead to superior outcomes compared to C-TKA.

## Patients and methods

### Patients

We conducted a retrospective, single-center study. Including all patients who underwent TKA for arthritis using the HLS Kneetec^TM^ Corin^®^ between 01/2022 and 01/2025 in our secondary care center. Each patient received a primary C-TKA in one knee and then an RA-TKA in the other knee. Patients who received a different implant or were operated on in another center were excluded.

**Figure 1:**
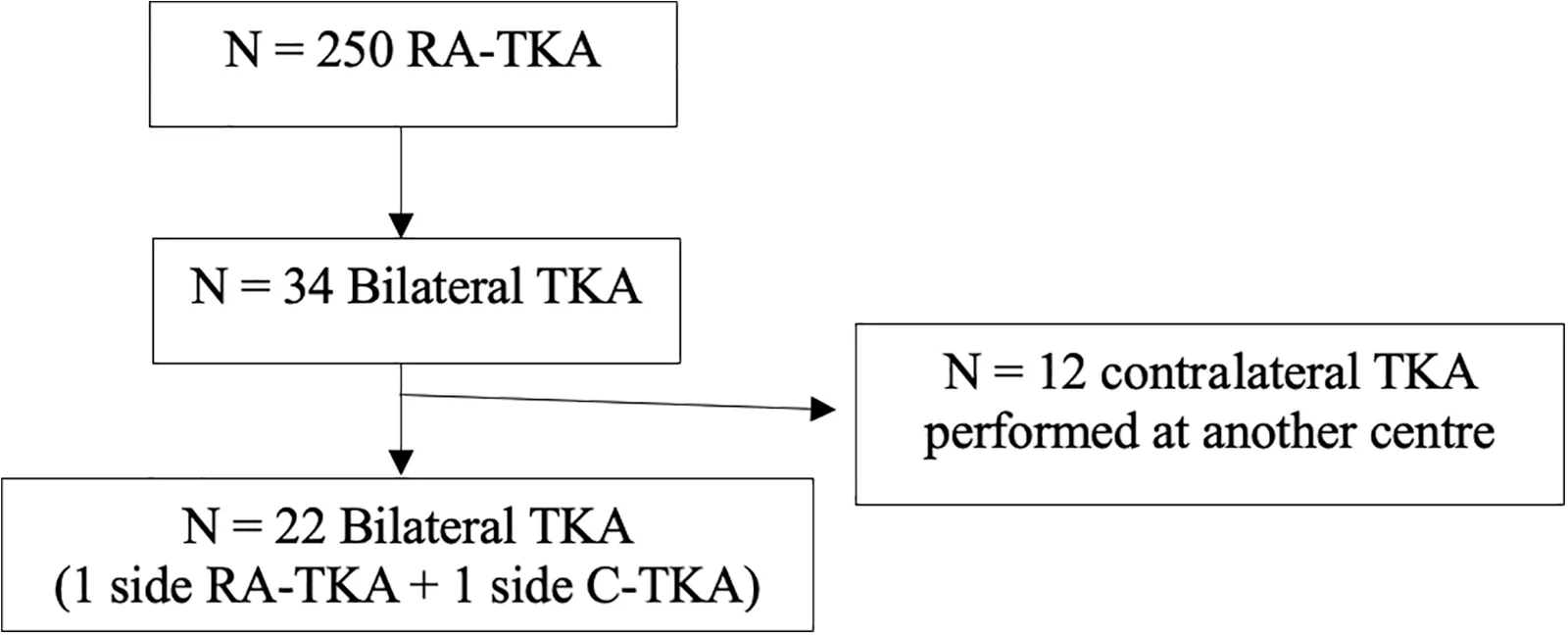
Flowchart.

A total of 44 knees from 22 patients were analyzed (cf. flowchart, Figure 1). 73% were women (*n* = 16/22) and 27% were men (*n* = 6/22). Mean age was 71.9 ± 7.4 years [55–81]. 10 (*n* = 4/44) of the prostheses were fully cemented (femur and tibia), while 90% (*n* = 40/44) were uncemented. The mean time between the two procedures was 22 ± 19 months [6–51]. All patients had a varus morphotype, with coronal plane alignment of the knee (CPAK) distributed as follows: 20% (*n* = 4/22) CPAK 1, 40% (*n* = 9/22) CPAK 4, and 40% (*n* = 9/22) CPAK 7 [8].

### Methods

All TKAs used the same implant (HLS Kneetec^TM^, a tri-condylar posterior-stabilized rotating-platform, available in cemented and cementless versions). All surgeries were performed in the same hospital by the same five senior surgeons. An AMA alignment technique [4] was used for every case. The implants were either cemented or cementless, as decided by the surgeon. The patella was systematically resurfaced, a medial parapatellar (trans-quadricipital) approach without tourniquet was used, intra-articular ropivacaine and tranexamic acid were administered, and all patients followed the same postoperative protocol, including immediate ambulation and early physiotherapy.

The AMA technique is a hybrid approach that combines conventional mechanical alignment on the tibial side, meaning the tibial component is implanted orthogonally (TMA = 90°). Ligament balancing is then achieved through femoral component positioning and, when needed, selective soft-tissue releases. The goal of this technique is to preserve the patient’s pre-arthritic limb phenotype by intentionally under-correcting the native varus or valgus deformity by up to 3°. The HLS Kneetec is designed so that the tibial component is implanted orthogonally; therefore, alternative alignment strategies, such as functional alignment [9, 10] cannot be performed.

In C-TKA, the tibial cut is performed first using an intramedullary rod set to the mechanical, i.e., 90° TMA with no posterior slope (0°). Valgus adjustment for the distal femoral cut is then performed using a manual ligament tensioner, which is also used to determine femoral rotation based on a posterior condyle reference. For varus knees, imbalance is corrected when needed by releasing the deep medial collateral ligament and the medial capsule. For valgus knees, the lateral collateral ligament is released, along with the iliotibial band and posterolateral capsule when required.

With the Corin RA-TKA system, which includes an imageless navigation platform (Omnibotic Work Station), a robotic ligament balancer (Balancebot^TM^), and a passive robotic femoral cutting guide (Omnibot), the hip, knee, and ankle centers are first registered, followed by bone morphing of the knee. The tibial cut is then performed first, using the navigated tibial cutting guide set orthogonally, i.e., 90° TMA with no posterior slope (0°). Then Balancebot^TM^ is used to record flexion–extension gaps throughout the full range of motion. Based on these measurements, bone resections and the femoral component position are adjusted in the 3D planning software to optimize soft-tissue balance. After finalizing the distalization, anteroposterior position, varus–valgus alignment, axial rotation, and flexion–extension of the femoral component on the Omnibotic Work Station, the femoral cut is performed using the Omnibot passive robotic cutting guide.

### Methods of assessment

Primary outcomes measured were limb alignment (hip–knee–ankle (HKA)), range of motion (ROM), and Forgotten joint score (FJS) one year postoperatively.

Secondary outcomes included operating time, hospital stay, incision length, blood loss (based on J1 hemoglobin), and radiographic evaluation (femoral mechanical angle (FMA), tibial mechanical angle (TMA), tibial slope angle (TSA), femoral axial femoral angle (FARA) (collected in operative report), and patellar tilt angle (PTA)).

### Statistical analysis

Statistical analysis was performed using an intra-individual model, with each patient serving as their own control (RA-TKA on one knee and C-TKA on the other). Normality was assessed using the Shapiro–Wilk test. Comparisons between the two techniques were made using the paired Student’s *t*-test for normally distributed data and the Wilcoxon signed-rank test for non-normally distributed data. Effect sizes were calculated using the intraclass correlation coefficient (ICC) or Cohen’s *d*. Correlation analyses between the different parameters were performed using Pearson’s or Spearman’s correlation tests. Statistical significance was set at *p* < 0.05. All analyses were conducted using R Studio (version 4.4.2, 2023.06.2+561).

## Results

The main results of this study are that RA-TKA resulted in a frontal limb alignment closer to the target of 180° ± 3° sought with AMA. Mean HKA was 177.9° ± 1.8° in RA-TKA groups vs. 175.3° ± 3.2° in C-TKA group (*p* = 0.012). RA TKA also resulted in 7° increase in range of motion (C-TKA 108° ± 18.57° [60°–120°] vs. RA-TKA 115° ± 12.28° [100°–130°], *p* = 0.032) and 13.9 points increase in FJS (89.1 ± 9.3 [60 – 100] vs. 75.2 ± 20.8 [50 – 100]*, p* < 0.001).

The analysis of secondary endpoints did not reveal any significant differences regarding hospital stay (*p* = 0.8), blood loss (*p* = 0.1), and incision length (*p* = 0.76), while RA-TKA operating time was 19.5 min longer (*p* = 0.004).

Regarding radiographic parameters, RA-TKA resulted in 1.3° less femoral valgus, as reflected by a lower FMA (*p* = 0.001). TMA and TSA did not differ significantly between groups (*p* = 0.70 and *p* = 0.11). Whereas FARA demonstrated 1.1° increased external rotation (*p* = 0.0006), Patellar tilt was similar between groups as reflected by PTA (*p* = 0.21).

There were no reoperations among these 44 knees.

All the results are presented in Table 1.

**Table 1:** Comparison of outcomes between C-TKA and RA-TKA.

	C‑TKA	RA‑TKA	*p*-value
HKA	175.3° ± 3.2 [172–179]	177.9° ± 1.8 [176–180]	**0.012**
ROM	108° ± 18.57 [60 – 120]	115° ± 12.28 [100–130]	**0.032**
FJS	75.2 pts ± 20.8 [50 – 100]	89.1 pts ± 9.3 [60 – 100]	**0.0001**
Operating time	75.9 min ± 17.4 [57–111]	95.4 min ± 21.1 [70–150]	**0.004**
Hospital stay	4.95 days ± 1.68 [2–8]	4.82 days ± 1.84 [3–8]	0.79
Incision length	18.03 cm ± 1.84 [14.49–21.09]	18.18 cm ± 2.68 [14.1–23.59]	0.76
Postoperative hemoglobin	12.15 g/dl ± 1.23 [10.1–14.6]	12.46 g/dl ± 1.18 [9.6–14.5]	0.1
FMA	88.4° ± 1.45 [85.9–91.9]	87.1° ± 0.87 [86.2–90.3]	**0.001**
TMA	89.76° ± 1.61 [86.9–93.9]	89.69° ± 0.99 [89.0–92.0]	0.70
TSA	88.51° ± 1.88 [84.0–92.0]	89.53° ± 2.90 [84.9–93.6]	0.11
FARA	2.5° ± 1.14 [1–4]	3.6° ± 2.04 [0–6]	**0.0006**
PTA	3.4° ± 3.2 [0–9.3]	2.3° ± 2.87 [0–8.7]	0.21

HKA: Hip-Knee-Ankle angle; ROM: Range of Motion; FJS: Forgotten Joint Score; FMA: Femoral Mechanical Angle; TMA: Tibial Mechanical Angle; TSA: Tibial Slope Angle; FARA: Femoral Axial Rotation Angle; PTA: Patellar Tilt Angle; C-TKA: Conventional Total Knee Arthroplasty; RA-TKA: Robotic-Assisted Total Knee Arthroplasty.Values are presented as mean ± SD [range].

## Discussion

This study demonstrated that the Omnibotics RA-TKA improves the precision in achieving the planned limb alignment. Robotic assistance may also provide benefits in terms of ROM and FJS. To our knowledge, it is the only study to evaluate AMA in patients undergoing bilateral TKA. Furthermore, few studies have investigated RA-TKA that utilizes a dynamic ligament balancer.

Although the OMNIBotics system has been the subject of relatively few publications, its clinical benefit remains debated. Pascal et al. [5], who evaluated the same OMNIBotics system reported significantly better Oxford Knee Scores and FJS (mean FJS 70.7 ± 22.5 in the RA-TKA group vs. 56.9 ± 22.7 in the C-TKA group; *p* = 0.002), as well as superior precision in reproducing the desired alignment. Their findings support that the OMNIBotics system enables accurate reproduction of the target alignment, improving patients’ functional prognosis at one year compared with conventional mechanical instrumentation.

In contrast, Vermue et al. [11] conducted a randomized study of 60 patients comparing RA-TKA with the BalanceBot standardized laxity assessment at 80 N versus conventional manual ligament tensioning. Unlike the retrospective series of Pascal et al. [5], and the present study, they found no clinically meaningful differences in functional outcomes, alignment, or soft-tissue balance at two-year follow-up. On this basis, the authors advised against routine use of the OMNIBotics system, citing its increased cost and longer operative time.

The present cohort yielded markedly higher FJS scores than those reported in the literature [5]: 89.1 ± 9.3 for RA-TKA and 75.2 ± 20.8 for C-TKA. This discrepancy may be partly explained by the inclusion of bilateral TKA patients, which introduces a recruitment bias towards individuals already satisfied with their first-knee procedure who subsequently opted for contralateral surgery. Importantly, the FJS was significantly higher in the RA-TKA group; however, the C-TKA group had already exceeded the minimal clinically important difference (MCID) threshold, which limits the clinical interpretation of the between-group difference. Furthermore, when considering the proportion of patients achieving the Patient Acceptable Symptom State (PASS), as reported by Vermue et al., no significant difference appears to be associated with the use of Omnibot in this series.

A noteworthy discordance was also observed between the excellent FJS values and the range of motion outcomes (115° ± 12.28°), which remained below the optimal flexion targets after TKA (128–132°) [12]. Pascal similarly reported flexion values below target with Omnibotics RA-TKA (117° ± 8°) [5].

Operative time was significantly longer in the RA-TKA group. This increase is attributable to several additional steps required in robotic surgery, including acquisition of the hip, knee, and ankle centers, accurate bone morphing, and pre-resection planning. Moreover, the assessment of ligament tension using the BalanceBot robotic ligament tensioner, rather than manual varus–valgus stress testing performed directly by the surgeon, further contributes to the additional operative time, as also reported by Vermue et al. [11].

The impact of the learning curve on operative time was not specifically evaluated in this study; however, it has already been established that increasing RA-TKA surgical experience reduces operative time without significantly affecting functional outcomes[13, 14].

Regarding blood loss, no significant difference could be demonstrated. Larger cohort studies have already shown that the use of robotic or navigation systems that do not violate the femoral and tibial canals helps reduce postoperative anemia and the need for transfusions [15].

A further limitation of the Omnibot device is that it can only be inserted after completion of an orthogonal tibial cut, making it incompatible with alignment philosophies that rely on preserving or reproducing joint-line obliquity. Despite that, it has been shown to yield superior outcomes [16, 17]. The latest evolution of the system, now available as the Apollo Knee (Corin), overcomes this limitation by allowing the use of a ligament tensioning device prior to the tibial cut.

This study has several important limitations that must be considered when interpreting the results.

This was a retrospective, single-center study with a limited sample size, which may have restricted statistical power.

- The RA-TKA was always performed as the second procedure (mean interval: 22 months after C-TKA). This systematic sequencing creates inseparable confounding between the technology effect (robotic vs. conventional) and several temporal effects:
  - The patient’s learning and rehabilitation effect, patients already familiar with postoperative protocols may achieve better ROM and FJS on their second surgery, regardless of technique.
  - Increased patient confidence during the second-sided procedure, a phenomenon already documented in the bilateral TKA literature [18].
  - The surgeon’s learning curve with the new robotic system. Consequently, the technology effect cannot be isolated from temporal effects in this design, which represents the most important threat to internal validity. Any observed difference in functional outcomes between the two techniques must therefore be interpreted with caution.
- The bilateral TKA design improves comparability between techniques by using each patient as their own control; however, it introduces a selection bias because only patients sufficiently satisfied with their first procedure (C-TKA) elected to undergo contralateral surgery. This enriches the cohort with patients predisposed to good outcomes, which likely amplifies the FJS values observed in both groups and limits generalisability to the general TKA population.
- Our series included only varus morphotypes, which does not reflect the full distribution of CPAK phenotypes in the general population [8]. Findings may not be generalizable to valgus or neutral knees. The sample size was insufficient to allow analysis of the impact of preoperative deformity on the outcomes.
- The relatively high number of operating surgeons compared with the total number of prostheses implanted introduces additional variability in surgical technique that could not be controlled for in the analysis.
- FJS scores in the C-TKA group were already above the MCID threshold, limiting the clinical relevance of the between-group difference despite its statistical significance. The high absolute FJS values in both groups further reflect the recruitment bias described above.

## Conclusion

In patients undergoing bilateral TKA with an adjusted mechanical alignment and a ligament-balanced approach, both RA-TKA and C-TKA reached the MCID. However, Omnibotics allowed more accurate execution of the planned alignment targets.

## Data Availability

Blinded Data and materials are available on request.
